# Italian guidelines for the management of irritable bowel syndrome in children and adolescents: Joint Consensus from the Italian Societies of: Gastroenterology, Hepatology and Pediatric Nutrition (SIGENP), Pediatrics (SIP), Gastroenterology and Endoscopy (SIGE) and Neurogastroenterology and Motility (SINGEM)

**DOI:** 10.1186/s13052-024-01607-y

**Published:** 2024-03-14

**Authors:** Giovanni Di Nardo, Giovanni Barbara, Osvaldo Borrelli, Cesare Cremon, Valentina Giorgio, Luigi Greco, Michele La Pietra, Giovanni Marasco, Licia Pensabene, Marisa Piccirillo, Claudio Romano, Silvia Salvatore, Michele Saviano, Vincenzo Stanghellini, Caterina Strisciuglio, Renato Tambucci, Rossella Turco, Letizia Zenzeri, Annamaria Staiano

**Affiliations:** 1 Department of Neurosciences, Mental Health and Sensory Organs (NESMOS), Faculty of Medicine and Psychology, Sapienza University of Rome, Pediatric Unit, Sant’Andrea University Hospital, Rome, Italy; 2 IRCCS Azienda Ospedaliero Universitaria di Bologna, Bologna, 40126 Italy; 3 Department of Medical and Surgical Sciences, University of Bologna, Bologna, 40126 Italy; 4 Neurogastroenterology & Motility Unit, Gastroenterology Department, Great Ormond Street Hospital for Children, London, UK; 5 Department of Woman and Child Health and Public Health, Fondazione Policlinico Universitario A. Gemelli IRCCS, Università Cattolica del Sacro Cuore, Rome, Italy; 6 General Pediatrician, Heath Care Agency of Bergamo, Bergamo, Italy; 7 General Pediatrician, Heath Care Agency of Naples, Naples, Italy; 8 Department of Medical and Surgical Sciences, Pediatric Unit, Magna Graecia University, Catanzaro, Italy; 9 Pediatric Gastroenterology and Cystic Fibrosis Unit, Department of Human Pathology in Adulthood and Childhood “G. Barresi”, University of Messina, Messina, Italy; 10 Pediatric Department, “F. Del Ponte” Hospital, University of Insubria, Varese, Italy; 11 Department of Woman, Child and General and Specialized Surgery, University of Campania “Luigi Vanvitelli”, Naples, Italy; 12 Digestive Endoscopy and Surgery Unit, Bambino Gesù Children’s Hospital, IRCCS, Rome, Italy; 13 Department of Pediatrics, Santobono-Pausilipon Children’s Hospital, Naples, Italy; 14 Department of Translational Medical Science, Section of Pediatrics, University Federico II, Via S. Pansini 5, Naples, 80131 Italy

**Keywords:** Irritable bowel syndrome, Children, Functional gastrointestinal disorders, Constipation, Diarrhea, Chronic abdominal pain

## Abstract

The irritable bowel syndrome (IBS) is a functional gastrointestinal disorder (FGID), whose prevalence has widely increased in pediatric population during the past two decades. The exact pathophysiological mechanism underlying IBS is still uncertain, thus resulting in challenging diagnosis and management. Experts from 4 Italian Societies participated in a Delphi consensus, searching medical literature and voting process on 22 statements on both diagnosis and management of IBS in children. Recommendations and levels of evidence were evaluated according to the grading of recommendations, assessment, development, and evaluation (GRADE) criteria. Consensus was reached for all statements. These guidelines suggest a positive diagnostic strategy within a symptom-based approach, comprehensive of psychological comorbidities assessment, alarm signs and symptoms’ exclusion, testing for celiac disease and, under specific circumstances, fecal calprotectin and C-reactive protein. Consensus also suggests to rule out constipation in case of therapeutic failure. Conversely, routine stool testing for enteric pathogens, testing for food allergy/intolerance or small intestinal bacterial overgrowth are not recommended. Colonoscopy is recommended only in patients with alarm features. Regarding treatment, the consensus strongly suggests a dietary approach, psychologically directed therapies and, in specific conditions, gut-brain neuromodulators, under specialist supervision. Conditional recommendation was provided for both probiotics and specific fibers supplementation. Polyethylene glycol achieved consensus recommendation for specific subtypes of IBS. Secretagogues and 5-HT4 agonists are not recommended in children with IBS-C. Certain complementary alternative therapies, antispasmodics and, in specific IBS subtypes, loperamide and rifaximin could be considered.

## Introduction

**Fig. 1:**
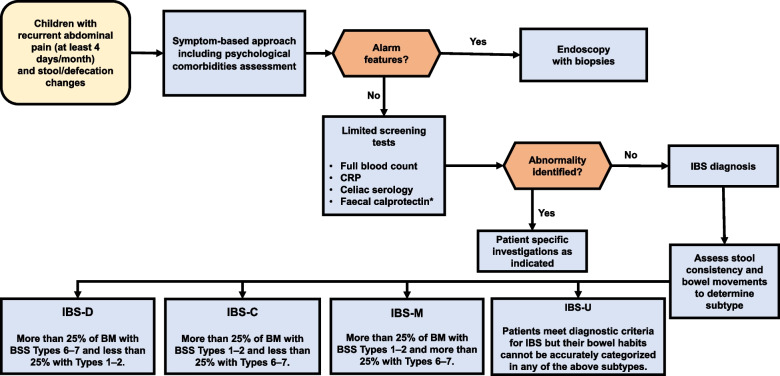
Diagnostic approach to children with Irritable Bowel Syndrome (IBS). Legend: *only in patients with IBS diarrhea subtype

**Fig. 2:**
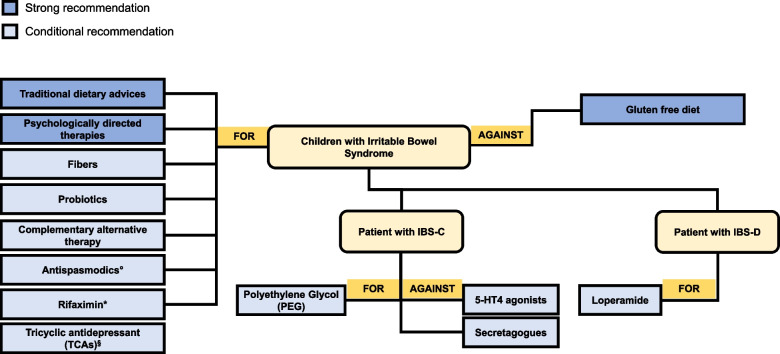
Therapeutic strategies in children with Irritable Bowel Syndrome (IBS). Legend: *in children with IBS without constipation in which other treatments have failed; °when other therapeutic strategies have failed; §in children with IBS in which other treatments have failed and only under specialist supervision

Irritable bowel syndrome (IBS) is one of the most common disorders of gut-brain interaction (DGBI) and its prevalence is increasing in the last decades within the pediatric population. Similarly to other DGBIs, IBS seems to result from the disruption of one or more elements part of the microbiota-gut-brain axis, in response to different triggering events on a background of a genetic predisposition [1, 2]. Due to a lack of specific biological markers, IBS is currently defined according to the symptom-based diagnostic criteria established by the Rome Foundation. The Rome criteria define diagnostic criteria for functional gastrointestinal disorders (FGIDs), are regularly updated and are currently on their 4th iteration [3]. The prevalence of IBS in children varies across studies and countries probably due to cultural differences in terms of pain characteristics and bowel habits. In Asia, a systematic review and meta-analysis on IBS showed a prevalence of 12.4% in children [4]. Several studies from Greece, Nigeria, South America and Sri Lanka have recognized IBS as the most prevalent DGBI among children and adolescents (2.9%, 9.9%, 3.8–6.4% and 3.6–7% respectively) [5–9]. Conversely, studies from United States and from the Mediterranean region have shown lower prevalence rates of IBS (2.8% and 4–5.1%, respectively) [10–12]. Regardless of prevalence, IBS has a significant impact on lives of affected children [13, 14] and their families and remains a challenge in terms of diagnosis and management for pediatricians [15, 16]. Multiple and unnecessary tests are frequently performed leading to a significant burden on national healthcare systems [17, 18]. To the best of our knowledge, only a few documents have specifically addressed this topic in the pediatric setting [19]. Therefore, a joint group of experts of the Italian Societies of Gastroenterology, Hepatology and Pediatric Nutrition (SIGENP), Pediatrics (SIP), Gastroenterology and Endoscopy (SIGE) and Neurogastroenterology and Motility (SINGEM), identified the need to provide clinicians with high quality evidence, when available, in order to answer essential questions related to the diagnosis (Fig. 1) and management (Fig. 2) of IBS in children.

## Methods

Selected members of the Italian Society of Gastroenterology, Hepatology and Pediatric Nutrition (SIGENP), Pediatrics (SIP), Gastroenterology and Endoscopy (SIGE) and Neurogastroenterology and Motility (SINGEM) participated to the Delphi process to develop consensus statements on the diagnosis and treatment of IBS in the pediatric population. The Delphi process is based on the principles of evidence-based medicine and consists of a systematic search of literature, a production of statements based on the best available evidence, and a voting process in order to determine consensus, especially for those fields of medicine not supported by evidences derived from controlled trials [20]. Each statement reported the quality of available evidence and the strength of the recommendation according to the Grading of Recommendations, Assessment, Development, and Evaluation (GRADE) system [21]. At the end of the Delphi process, Vincenzo Stanghellini and Carlo Di Lorenzo revised the statements, the supporting evidences, and the strength of recommendations as external reviewers.

The Core Working Group, composed by 7 panel members (GDN, LZ, GM, CC, AS, CR and GB) with expertise in IBS and/or Delphi consensus processes, identified 22 clinical questions to answer using the patient, intervention, control, and outcome (PICO) process (Table 1). The Italian Consensus Group was recruited within the SIGENP and other Italian societies in the field of Gastroenterology and Pediatrics and included experts in IBS. All members submitted a conflict-of-interest statement by February 2023. All panel members performed a systematic literature review to answer each PICO and drafted statements with a summary of evidence. Grading of the strength of recommendation was performed using accepted criteria and, finally, one to two rounds of repeated voting of the statements were performed in order to reach consensus.

**Table 1:** All PICO and statements with endorsement, level of evidence, grade of recommendation and agreement

**PICO/Statement number**	**PICO**	**Statement**	**Endorsement**	**Level of evidence**	**Strength of recommendation**	**Agreement**
**Diagnosis**						
1.1	Are the clinical history and symptoms required for IBS diagnosis in children?	We recommend the assessment of patient’s symptoms and clinical history for diagnosis and management of children with IBS	Yes	NA	Consensus	100%
1.2	Should children with IBS diagnosis be regularly evaluated for psychological comorbidities?	We recommend psychological comorbidities assessment in children with IBS	Yes	NA	Consensus	100%
1.3	Is it more appropriate to approach children with suspected IBS using a positive diagnostic approach as opposed to one of exclusion?	We recommend a positive diagnostic strategy in children with symptoms suggestive of IBS	Yes	NA	Consensus	100%
1.4	Should all children with a diagnosis of IBS be evaluated for occult constipation?	We recommend to rule out occult constipation in children with symptoms suggestive of IBS when therapeutic strategies have failed	Yes	NA	Consensus	100%
1.5	Should children with IBS symptoms be tested for celiac disease (CD)?	We recommend serologic testing for CD in all children with IBS symptoms	Yes	Moderate	Strong	100%
1.6	Can fecal calprotectin, and/or CRP be used to rule out IBD in children with IBS symptoms?	We recommend the use of fecal calprotectin^1^ and C-reactive protein^2^ to exclude inflammatory bowel disease in patients with IBS symptoms and diarrhea without alarm features	Yes	^1^Very low ^2^Very low	^1^Strong ^2^Conditional	94.4%
1.7	Should IBS patients be routinely checked for stool pathogens?	We recommend against routine stool testing for enteric pathogens in children with IBS	Yes	Low	Conditional	100%
1.8	When is colonoscopy indicated in patients with IBS symptoms?	We recommend colonoscopy only in patients with IBS symptoms and alarm features	Yes	NA	Consensus	100%
1.9	Should patients be tested for food allergy/intolerance?	We recommend against testing for food allergy/intolerance in children with IBS	Yes	NA	Conditional	100%
1.10	Should patients be tested for SIBO?	We recommend against routine testing for small intestinal bacterial overgrowth in children with IBS symptoms	Yes	Very Low	Strong	100%
**Treatment**						
2.1	Should dietary approaches be used in children with IBS?	We recommend traditional dietary advices as a first line dietary approach^1^. A gluten free diet is not recommended in patients with IBS^2^	Yes	^1^Very low ^2^Very low	^1^Strong ^2^Strong	100%
2.2	Should fiber be used to treat global IBS symptoms in children?	We recommend certain fibers supplementation to treat abdominal pain in children with IBS	Yes	Moderate	Conditional	88.9%
2.3	Should probiotics be used to treat global IBS symptoms in children?	We recommend the use of certain probiotic strains to treat global IBS symptoms	Yes	Moderate	Conditional	88.9%
2.4	Should polyethylene glycol be recommended to treat constipation in children with IBS-C?	We recommend to use PEG to treat constipation in children with IBS-C	Yes	NA	Consensus	100%
2.5	Should secretagogues be used to treat IBS-C symptoms in children?	We recommend against the use of intestinal secretagogues for the treatment of pediatric IBS-C	Yes	NA	Consensus	94.4%
2.6	Should 5-HT4 agonists be used to treat IBS-C symptoms?	We suggest against the use of 5-HT4 agonists in pediatric patients with IBS-C	Yes	Low	Conditional	100%
2.7	Should rifaximin be used to treat global IBS symptoms?	The use of rifaximin could be considered in children with IBS without constipation in which other treatments have failed	Yes	Very low	Consensus	94.5%
2.8	Should loperamide be used to treat IBS-D symptoms?	We recommend the use of loperamide to manage diarrhea in IBS-D, although its chronic use must be avoided	Yes	NA	Consensus	100%
2.9	Should antispasmodics be used to treat global IBS symptoms?	The use of antispasmodics could be considered for global symptom improvement in children with IBS when other therapeutic strategies have failed	Yes	Very low	Consensus	100%
2.10	Should gut-brain neuromodulators be used to treat IBS symptoms?	The use of gut-brain neuromodulators, under specialist supervision, could be considered to treat severe abdominal pain in children with IBS in which other treatments have failed	Yes	Moderate	Strong	94.4%
2.11	Should complementary alternative therapies be used to treat IBS symptoms?	The use of certain complementary alternative therapies could be considered to treat IBS symptoms	Yes	Very low	Conditional	100%
2.12	Should psychologically directed therapies be used to treat global IBS symptoms?	We strongly recommend the use of psychologically directed therapies for the treatment of global symptoms	Yes	Low	Strong	94.4%

*Abbreviations: PICO* Patient, Intervention, Control, Outcome, *NA *Not Available: unable to assess using GRADE methodology, *IBS *Irritable Bowel Syndrome, *IBD* Inflammatory Bowel Disease, *CD* Celiac Disease, *CRP* C-Reactive Protein, *SIBO* Small Intestinal Bacterial Overgrowth, *PEG* Polyethylene Glycol, *IBS-C* Irritable Bowel Syndrome with Constipation, *5-HT4* 5- Hydroxytryptamine-4, *IBS-D* Irritable Bowel Syndrome with Diarrhea

The literature search was performed using MEDLINE, EMBASE, Web of Science and the Cochrane Database of Systematic Reviews until March 30th 2023, without time and/or language restrictions. References were available on an online shared folder accessible to all members.

Researchers prioritized data from systematic reviews and meta-analyses of randomized controlled trials (RCTs) and data regarding the specific Italian population in order to identify evidence related to that specific subgroup, when available.

The strength of recommendation (SOR) was assessed using the GRADE methodology (https://www.gradeworkinggroup.org/) and the recommendations for the different clinical scenarios were classified into three categories: strong (desirable effects outweigh undesirable effects), conditional (trade-offs are less certain) or consensus (the expert opinion supports the guideline recommendation even though the available scientific evidence did not provide consistent results or controlled trials were lacking).

To evaluate the levels of evidence (LoE), the following definitions were used: high (further research is unlikely to change confidence in the estimate), moderate (further research is likely to change confidence in the estimate), low (further research is very likely to change confidence in the estimate), or very low (the estimate of the effect is very uncertain). The level of evidence could be downgraded or upgraded according to different factors such as limitations or implementations in the study design, imprecision of estimates, variability in the results, indirectness of the evidence, publication bias, large magnitude of effects, dose–response gradient, or if all the plausible biases would reduce an apparent treatment effect. In addition, the recommendations also considered other factors as alternative management strategies, variability in values and preferences and the costs.

The finalized list of statements with the summary of evidence was edited and discussed in a 3-day telematic session. Thereafter, all participants were asked to participate in a first blinded voting round in May 2023 to vote on their agreement with statements using a 6-point Likert scale (Table 2) and to provide feedback on their clarity. When 80% of the Consensus Group agreed with a statement (A + or A), this was defined as consensus. The agreement on all statements was reached after the first voting round (summarized in Table 1) except for PICO 18 and 21 which were revised and then approved after the second voting round. Subsequently, the manuscript was drafted and reviewed by participants for final approval. The final document was then submitted for external review to improve the quality of the guidelines.

**Table 2:** Six-point Likert scale

**Point**	**Description**
A +	agree strongly
A	agree with minor reservation
A-	agree with major reservation
D-	disagree with major reservation
D	disagree with minor reservation
D +	disagree strongly

## Diagnosis

PICO 1: Are the clinical history and symptoms required for IBS diagnosis in children?

Statement: We recommend the assessment of patient’s symptoms and clinical history for diagnosis and management of children with IBS.

Statement endorsed, overall agreement: 100%: A + 100%, A 0%, A- 0%, D- 0%, D 0%, D + 0%.

LoE: unable to assess using GRADE methodology; SOR: consensus recommendation.

Summary of evidence: IBS is a clinical diagnosis. The Rome IV criteria currently provide symptom-based guidelines that can be used to diagnose children and adolescents with IBS [3]. They are focused on a careful history and physical examination that can help to identify this disorder and to establish a positive diagnosis of IBS in children, in the absence of alarm signs and symptoms. Alarm symptoms include pain disrupting sleep or localized in right upper or right lower quadrant, rectal bleeding, fever, weight loss, family history of inflammatory bowel disease or celiac disease, low hemoglobin level.

The clinical history should focus on the characteristics of abdominal pain, on changes in pain intensity related to bowel motions and on details about stool patterns.

Symptoms must be recurrent and should occur at least four times a month for a minimum of 2 months. Symptoms such as abdominal bloating and defecation urgency can also occur [22].

Symptoms can often appear after a gastrointestinal infection [23, 24] or after emotional distress [25] and patients may also experience increased sadness, interpersonal sensitivity and sleep disturbances [26]. Somatic symptoms and psychological problems, including anxiety and depression, are also commonly found in children with IBS [27] and their identification may aid in formulating the diagnosis and starting correct treatment of these children.

The Rome IV criteria identify different subtypes of IBS according to the stool consistency on the days with abnormal bowel movements and include IBS-C (constipation), IBS-D (diarrhea), IBS-M (mixed) and IBS-U (unsubtyped) [14, 28]. Patients with IBS-C have > 25% of their bowel movements associated with stool types 1 or 2 according to Bristol stool form scale (BSFS), while those with IBS-D have > 25% of their bowel movements associated with stool types 6 or 7. Patients with IBS-M have > 25% of their bowel movements associated with stool types 1 or 2 and > 25% of their bowel movements associated with stool types 6 or 7 [29]. The clinical subtypes should not be considered a rigid classification, as they could change over time, but the classification could help physicians to costumize from time to time management in children with IBS.

PICO 2: Should children with IBS diagnosis be regularly evaluated for psychological comorbidities?

Statement: We recommend psychological comorbidities assessment in children with IBS.

Statement endorsed, overall agreement: 100%: A + 94.4%, A 5.6%, A- 0%, D- 0%, D 0%, D + 0%.

LoE: unable to assess using GRADE methodology; SOR: consensus recommendation.

Summary of evidence: Several studies have reported increased anxiety and depression in children with IBS compared to healthy children [30–35]*.* Similarly to adults, whether abdominal pain or mental symptoms come first remains to be elucidated also in children [36–39]. Anxiety and depression appear to be associated with increased severity of abdominal pain and disability [40–43], although other studies did not confirm these data [44]. Additionally, several studies have shown that the association between psychological comorbidity and childhood abdominal pain increases the risk of having an IBS disorder in adulthood [36, 45–47].

Although anxiety and depression are widely studied, they may not be the most important factors in children with IBS. Holler and colleagues observed that somatization and pain catastrophizing mediate the association between anxiety/depression and the severity of IBS abdominal pain in children [27]. Previously, other studies have shown that children with functional abdominal pain had higher somatization scores compared to healthy children [48–52] and that this is more relevant for children with IBS than other functional abdominal disorders [50, 53]. In addition, Song and colleagues found that Korean children with IBS presented higher scores of stress compared to healthy children [54], a finding confirmed also by other authors [55]. Several studies have reported that functional gastrointestinal symptoms are significantly more common in children with a history of physical, emotional, and sexual abuse [56–58].

PICO 3: Is it more appropriate to approach children with suspected IBS using a positive diagnostic approach as opposed to one of exclusion?

Statement: We recommend a positive diagnostic strategy in children with symptoms suggestive of IBS.

Statement endorsed, overall agreement: 100%: A + 72.2%, A 27.8%, A- 0%, D- 0%, D 0%, D + 0%.

LoE: unable to assess using GRADE methodology; SOR: consensus recommendation.

Summary of evidence: The diagnosis of IBS is known to have a major impact on national healthcare systems and economies [17]. The estimated annual cost of caring for children with IBS in Europe is over 15 billion euros [18].

As stated in the Rome IV criteria [3], IBS is a functional bowel disorder in which recurrent abdominal pain is related to defecation or associated with change in bowel habits (frequency or appearance of stool). Symptom onset should occur at least 6 months prior to diagnosis and symptoms should be present on average at least 1 day per week during the last 3 months. Thus, IBS should be a positive diagnosis, based on medical interview, physical examination, and limited blood tests, and not the result of an exclusion algorithm aimed at ruling out all possible organic diseases. The decision to perform additional diagnostic procedures should be assessed individually. Velasco-Benítez and colleagues [59] demonstrated a sensitivity of 75% and a specificity of 90% with positive and negative predictive value of 85.8% and 79.9%, respectively for IBS diagnosis in children. Previously, also Miele and colleagues [60] had shown that the application of the Rome II criteria for functional gastrointestinal disease has a significant positive impact in reducing unnecessary gastrointestinal endoscopy in children.

Taken together, these considerations suggest that a positive approach is effective to reduce the cost of assessing IBS in children.

PICO 4: Should all children with a diagnosis of IBS be evaluated for occult constipation?

Statement: We recommend to rule out occult constipation in children with symptoms suggestive of IBS when therapeutic strategies have failed.

Statement endorsed, overall agreement: 100%: A + 72.2%, A 27.8%, A- 0%, D- 0%, D 0%, D + 0%.

LoE: unable to assess using GRADE methodology; SOR: consensus recommendation.

Summary of evidence: Constipation is a common condition in the pediatric population. Occult constipation is defined as a clinical condition with no obvious complaints of constipation and no symptoms suggestive of constipation, but at least one of the following: 1) hard stool consistency (stone or pellet-like) on rectal examination and 2) evidence of colon distended by feces on plain abdominal x-ray. Stool retention has been suggested as the cause of recurrent abdominal pain in children [61]. However, there are only two studies on this topic in children with IBS.

Pelvic floor function using anorectal manometry and balloon ejection test was tested in 67 adolescents with functional constipation (*n* = 16), fecal incontinence (*n* = 18), and IBS-C (*n* = 33) [62]. Patients classified as IBS-C were more likely to report weight loss (*p* = 0.03), bloating (*p* = 0.04), and incomplete rectal evacuation (*p* = 0.03), as compared to the other two groups. Furthermore, the test showed the presence of dyssynergy defecation in children with IBS-C.

Tosto and colleagues prospectively enrolled 26 consecutive children who meet Rome IV criteria for a diagnosis of IBS-D and IBS -M [63]. Patients who fulfilled criteria for suspect “occult constipation” received a bowel cleaning regimen with polyethylene glycol 3350 and were followed up for at least 6 months. 16 additional patients with IBS-C referred in the same period were enrolled as control. The endpoints were a decrease of more than 50% in abdominal pain intensity and frequency scores and resolution of diarrhea for patients with IBS-D and IBS-M. The endpoints were met by 8 (80%) and 14 (87%) of the patients with IBS-D and IBS-M, respectively, with decrease of abdominal pain and resolution of “diarrhea” (pseudo-diarrhea). The response was not significantly different from that observed in 15 (93%) of the IBS-C control group. Despite the small number of patients and the uncontrolled nature of the study, it suggests that a number of patients labeled as IBS-D or IBS-M may present functional constipation and should be managed as such.

PICO 5: Should children with IBS symptoms be tested for celiac disease (CD)?

Statement: We recommend serologic testing for CD in all children with IBS symptoms.

Statement endorsed, overall agreement: 100%: A + 88.9%, A 11.1%, A- 0%, D- 0%, D 0%, D + 0%.

LoE: Moderate; SOR: Strong.

Summary of evidence: IBS and Celiac Disease (CD) could present with similar manifestations in children, thus resulting in misdiagnosis. Hence, Rome IV criteria suggest an evaluation of CD in case of IBS-D [3]. Moreover, in central Europe, abdominal pain has been recently reported as the leading symptom in children with CD (in 33.3% of symptomatic children, and among those, in 66.4% of polysymptomatic children) followed by abdominal distension and diarrhea (56.7% and 54.2%, respectively) [64].

However, it is still unclear if children with IBS are more likely to have CD [65]. Cristofori and colleagues [66] in a 6-years prospective cohort study observed that 12 of 270 patients with IBS (4.45%) were positive for CD testing. Conversely, other authors failed in finding an association between recurrent abdominal pain and the prevalence of anti-endomysial antibody when compared to asymptomatic controls [67]. Falcon and colleagues reported that only 1 of 181 children with functional abdominal pain disorders (FAPDs) (0/84 with IBS) had positive CD serological testing, questioning the need for CD testing in all children with IBS [68]*.*

However, the prevalence of CD in Europe is higher compared to other countries [69] and in Italy it has been recently reported as one of the highest in world in school-age children [70].

Therefore, taking into account the significant potential consequences of missing the diagnosis of CD, we recommend serologic testing for CD with quantitative IgA levels and IgA anti-tissue transglutaminase (tTG) in all children with IBS symptoms if CD prevalence in the population is > 1% (as it is in Italy).

PICO 6: Can fecal calprotectin, and/or CRP be used to rule out IBD in children with IBS symptoms?

Statement: We recommend the use of fecal calprotectin^1^ and C-reactive protein^2^ to exclude inflammatory bowel disease in patients with IBS symptoms and diarrhea without alarm features.

Statement endorsed, overall agreement: 94.4%: A + 72.2%, A 22.2%, A- 5.6%, D- 0%, D 0%, D + 0%.

^1^ LoE: Very low; SOR: Strong. ^2^ LoE: Very low; SOR: Conditional.

Summary of evidence: In the last decade, the use of fecal calprotectin (FC) as a non-invasive screening method to screen for intestinal mucosal inflammation has increased both in children and adults [71]. A recent systematic review, analyzing 8 pediatric studies, concluded that fecal calprotectin is a valuable test to exclude IBD and to avoid invasive investigations, with particular reference to colonoscopy [72]. In keeping with these data, Heida and colleagues demonstrated that children should not undergo endoscopy when FC levels are < 50 mg/g [73]. A flowchart providing a guideline on how to proceed with a child presenting with gastrointestinal (GI) symptoms according to FC levels has been suggested [74]: in absence of “red flag” symptoms and FC < 250 μg/g in two separate samples, IBD will be unlikely and further investigations should not be performed [74]; instead, FC levels of > 250 μg/g in two separate samples in children with GI symptoms suggestive of IBD support the need for further invasive procedures [74]*.* Recently, a study in 853 children showed that fecal calprotectin had a fair accuracy, superior to C-reactive protein (CRP), hemoglobin levels and erythrocyte sedimentation rate (ERS) to discriminate between organic and functional causes of abdominal pain [75].

Thus, the ESPGHAN expert group recommended using the fecal calprotectin as a tool to differentiate functional abdominal pain disorders from organic diseases [76].

If low values of FC can exclude IBD with sufficient accuracy, high values do not exclude IBS, since increased levels of fecal calprotectin can also be found in children with IBS compared to healthy controls [77–79]. These data indirectly confirm the presence of a low-grade inflammation also in children with IBS. Similarly, it has been reported that median high-sensitive CRP levels in the IBS group were significantly higher than in healthy controls (1.80, IQR 0.7–4.04 mg/l vs 1.20, IQR 0.5–2.97 mg/l respectively, *p* < 0.006,) with the highest levels in IBS-D patients showing greater symptoms severity [80]*.*

Therefore, in patients with high levels of FC, IBS cannot be excluded and further examination could be required, whereas in patients with low levels of FC, IBD can be reasonably excluded.

PICO 7: Should IBS patients be routinely checked for stool pathogens?

Statement: We recommend against routine stool testing for enteric pathogens in children with IBS.

Statement endorsed, overall agreement: 100%: A + 88.9%, A 11.1%, A- 0%, D- 0%, D 0%, D + 0%.

LoE: Low; SOR: Conditional.

Summary of evidence: Zeevenhooven and colleagues [81] demonstrated that testing for Giardia lamblia in association to fecal calprotectin and celiac disease serology could have a high sensitivity and specificity in discriminating between organic and functional causes of chronic abdominal pain. Moreover, it has been suggested that in some countries such as Egypt, Pakistan, Turkey, and Poland, the prevalence of parasitic infections may justify stool testing in children with recurrent abdominal pain [82–86]. However, in developed countries, the prevalence of parasitic infections did not differ significantly in children with and without chronic abdominal pain [87, 88]*.* Even if there is the possibility to have post-infection IBS in children [23, 24, 89–92]*,* routine testing for enteric pathogens is not recommended in children with suspected IBS.

PICO 8: When is colonoscopy indicated in patients with IBS symptoms?

Statement: We recommend colonoscopy only in patients with IBS symptoms and alarm features.

Statement endorsed, overall agreement: 100%: A + 83.3%, A 16.7%, A- 0%, D- 0%, D 0%, D + 0%.

LoE: unable to assess using GRADE methodology; SOR: consensus recommendation.

Summary of evidence: The Rome IV criteria and the ESPGHAN and ESGE guidelines suggest to perform colonoscopy only if alarm signs and symptoms for organic intestinal diseases exist [93, 94]. Among alarm symptoms and signs for organic diseases in children with chronic abdominal pain [3, 95] the classic “triad” of bloody diarrhea, weight loss, and/or positive serum inflammatory markers (CRP and/or ESR) [96], and/or high levels of fecal calprotectin were found to present possible benefits from endoscopy [97]. The clinical value of colonoscopy in children with symptoms suggestive of IBS cannot be determined since the studies on this field are generically related to chronic abdominal pain [98–100].

Some studies focusing on the presence of intestinal low-grade inflammation in patients with FGIDs have found increased inflammatory cells (e.g. eosinophils in the duodenum for functional dyspepsia and mast cells in the ileum or colon for IBS) [78, 79, 101–104]. However, the clinical value of those findings is limited because their detection did not influence patients’ management.

In clinical practice, a common justification for performing endoscopies in children with functional abdominal pain disorders is the need for parents reassurance by demonstrating normal findings on the procedure [105], even if it carries risks related to the invasive nature of the procedure and anesthesia. However, Bonilla and colleagues [106] showed that the outcome of children with abdominal pain who underwent a negative endoscopy was similar to children with abdominal pain who did not undergo endoscopy.

PICO 9: Should patients be tested for food allergy/intolerance?

Statement: We recommend against testing for food allergy/intolerance in children with IBS.

Statement endorsed, overall agreement: 100%: A + 72.2%, A 27.8%, A- 0%, D- 0%, D 0%, D + 0%.

LoE: unable to assess using GRADE methodology; SOR: Conditional.

Summary of evidence: According to Rome IV criteria, the diagnosis of IBS is symptom-based, and patients who fulfill criteria for IBS without other signs and symptoms suspicious for food allergy should not be tested for food allergies or intolerances.

IBS shares pathophysiologic features with allergies, including both immune and psychological patterns, even though the mechanism through which allergens might play a role in the determinism of IBS remains unclear. In the absence of systemic allergic symptoms or oral allergy syndrome, allergic triggers for IBS are unlikely to be identified by standard testing [107].

Indeed, evidences regarding a possible link between IBS and allergic diseases are controversial [108]. Some authors observed that children with asthma have a significantly higher IBS prevalence [109–111], while others failed to find such an association [112, 113]. Most studies, however, were cross-sectional [112, 114–117] and did not use current diagnostic criteria for IBS. Among them, only Kumari and Colman used Rome III criteria. Three longitudinal studies were identified [118–120] and all reported a positive association between asthma and IBS.

A recent large prospective population-based birth cohort study [121] using Rome IV criteria, found age specific associations of asthma and food hypersensitivity at 12 and 16 years old with an increased risk for IBS in the latter group. The relative risk for IBS at 16 years increased with increasing number of concurrent allergy-related diseases, but the linear trend for relative risk was only borderline significant (*p* = 0.05).

Concerning food allergies and hypersensitivity, eczema, allergic rhinitis and dermatitis, several authors have described an association with IBS, postulating that children with antecedent allergic diseases have an increased risk of IBS compared to healthy population [110, 117, 118, 122]. However, most of these studies were based on selected samples, and used outdated or non-accepted criteria to define IBS, therefore no definitive conclusion can be drawn.

PICO 10: Should patients be tested for SIBO?

Statement: We recommend against routine testing for small intestinal bacterial overgrowth in children with IBS symptoms.

Statement endorsed, overall agreement: 100%: A + 94.4%, A 5.6%, A- 0%, D- 0%, D 0%, D + 0%.

LoE: Very low; SOR: Strong.

Summary of evidence: Two studies on small intestinal bacterial overgrowth (SIBO) in pediatric IBS have been published.

A case–control study assessed the prevalence of SIBO in 43 consecutive Italian children fulfilling Rome II criteria for IBS [123]. All subjects underwent lactulose/methane breath test (LBT) to diagnose SIBO. The prevalence of SIBO was 65% (28/43 children), and was significantly higher in patients with IBS in comparison with control subjects (7%, 4/56; OR 3.9, 95% CI 7.3–80.1, *p* < .00001). Patients with SIBO showed a trend toward a worse visual analogue scale (VAS) pain score compared to controls.

A prospective cohort study was performed on 161 Dutch children fulfilling the Rome III criteria for abdominal-pain-related FGIDs [124]. SIBO prevalence was assessed using glucose hydrogen breath test (GHBT). SIBO was diagnosed in 14.3% children (23/161); among those 69% (16/23) had IBS. SIBO positive patients more frequently complained of altered defecation patterns, loss of appetite and belching, which seemed to be predictors for SIBO.

So far, there is still insufficient evidence to justify the routine exclusion of SIBO in children with IBS.

PICO 11: Should dietary approaches be used in children with IBS?

Statement: We recommend traditional dietary advices as a first line dietary approach^1^*.* A gluten free diet is not recommended in patients with IBS^2^.

Statement endorsed, overall agreement: 100%: A + 88.9%, A 11.1%, A- 0%, D- 0%, D 0%, D + 0%.

^1^ LoE: Very low; SOR: Strong. ^2^ LoE: Very low; SOR: Strong.

Summary of evidence: A randomized double-blind, crossover trial evaluated the effect of a low FODMAPs diet on 33 children fulfilling Rome III criteria for IBS [125]. After 1-week baseline period, children were randomized to receive either a low fermentable oligo-, di- and monosaccharides, and polyols (FODMAPs) diet (containing 0.15 g/kg/day—maximum 9 g/day—of FODMAPs) or a typical American childhood diet [(TACD) containing 0.7 g/kg/day—maximum 50 g/day- of FODMAPs]. This dietary intervention lasted 48 h and was followed by a 5-day washout period, before crossing over to the other dietary intervention. Compared to baseline, children had fewer daily abdominal pain episodes during the low FODAMPs diet than during the TACD (*p* < 0.01).

Chumpitazi and colleagues [126] conducted a randomized controlled cross-over trial to assess whether supplementation of 0.5 mg/kg/day (up to 19 g) of inulin-fructo-oligosaccharide for 72 h worsened abdominal pain compared with placebo (maltodextrin) in 23 pediatric patients with IBS who were already on a low-FODMAP diet. Authors found that children had more episodes of abdominal pain/day during the fructan-containing diet vs the maltodextrin-containing diet. In particular, the frequency of abdominal pain was significantly higher during fructan supplementation vs placebo supplementation (*p* < 0.01). About 52% of children had a ≥ 30% increase in abdominal pain frequency after supplementation and were considered fructan sensitive.

Although these promising data suggest that low FODMAPs diet may be effective in reducing IBS symptoms in children, conflicted data have also been published. Another randomized study [127] evaluated the effect of a 2-months low FODMAPs diet on 60 children with IBS, fulfilling Rome IV criteria. Children were randomized to receive either a low FODMAP diet or a standard diet (i.e. a diet providing age-appropriate proteins, calories, vitamins, and mineral intake). At 2 months, patients were asked to score abdominal pain using the VAS scale, while their clinical status was assessed by clinicians using the Clinical Global Impression Improvement (CGI-I) scale. VAS score was significantly lower in the low-FODMAP diet group (*p* = 0.0001). Two months after the discontinuation of the intervention, both VAS and GGI-I were worse in the low-FODMAP compared to the standard diet group, suggesting that benefits from a low-FODMAP diet are not sustained in the long term in children with IBS.

Other potential limitations and concerns regarding this diet also include nutritional adequacy, costs and the risk of such food restriction to trigger disordered eating in adolescents. Additional studies are needed to clarify which children would benefit from a low FODMAPs diet and to evaluate its efficacy and compliance on long-term treatment.

One double-blind placebo-controlled gluten challenge in children with chronic functional GI symptoms was published [128]. A group of 1,114 children with functional gastrointestinal symptoms (i.e. chronic abdominal pain, diarrhea, bloating, dyspeptic symptoms diagnosed based on Rome III criteria) were screened to evaluate correlation between symptoms and gluten ingestion. Among them 96.7% did not show any correlation between symptoms and gluten ingestion and were excluded. The remaining 36 children underwent a 3-phases trial: (*i*) a phase of 2-weeks run-in, consisting in the exposure to a gluten-containing diet for baseline evaluation—in 5 children symptoms improved and were excluded; (*ii*) an open 2-weeks gluten-free diet (GFD) phase—3 children did not respond and were excluded from the subsequent phase; (*iii*) a placebo-controlled crossover trial after 1 week of washout from the GFD, in which 28 children were included. All children received one sachet per day either with a placebo or with gluten (10 g of gluten). At the end of the trial, the authors reported a decrease in all clinical scores during the open GFD, with 11/28 (39.2%) children having a global VAS improvement. However, no difference in the global VAS score was found during the blind administration of gluten or placebo, and 4/36 (14.3%) experienced the worsening of symptoms during the placebo administration.

A prospective, cross-sectional case-controlled study on 100 children with IBS according to Rome IV criteria, evaluated the effects of a Mediterranean diet (MD) on symptoms and quality of life [129]. Patients were divided into 2 groups: group 1 received a Mediterranean diet with good adherence (KIDMED Score ≥ 8 points [130, 131], and group 2 received a regular diet following local diet habit (Egypt). After 6 months, there was a significant improvement in all IBS scores in group I compared to group II. IBS symptoms severity score (IBS-SSS) and mean IBS-Quality of Life (QoL) in MD group significantly improved over the study period (*p* < 0.001).

In conclusion, based on a recent systematic review of intervention studies [132], low-FODMAP diet, fructose- or lactose-restricted diet (FRD or LRD) and GFD have no place in daily clinical practice for the management of children and adolescents with FGIDs. Nevertheless, some patients with IBS may experience some benefit from following a low-FODMAP diet or FRD/LRD [133]. Limited data suggest that MD may be promising in the management of FGIDs, especially in IBS patients, but more data are required to investigate the mechanisms of its protective effects. Based on the panelists’ expert opinion we suggest traditional dietary advices, consisting of regular meals, adjustment of fiber and fluid intake, decreasing sugar, fat and spicy meals intake, as first line dietary approach (https://www.efsa.europa.eu/sites/default/files/assets/DRV_Summary_tables_jan_17.pdf) in children with IBS.

PICO 12: Should fiber be used to treat global IBS symptoms in children?

Statement: We recommend certain fibers supplementation to treat abdominal pain in children with IBS.

Statement endorsed, overall agreement: 88.9%: A + 27.8%, A 61.1%, A- 5.6%, D- 5.6%, D 0%, D + 0%.

LoE: Moderate; SOR: Conditional.

Summary of evidence: In a 4-week randomized, double-blind pilot study, Romano and colleagues. evaluated the effects of partially hydrolyzed guar gum (PHGG) supplementation in 60 children with chronic abdominal pain or IBS. Children in the treatment group were provided 5 g/day of PHGG, mixed in fruit-juice, whereas the placebo group received only fruit-juice. In the IBS group, children supplemented with PHGG reported a significant reduction of clinical symptoms compared to the control group (43% vs 5%, *p* = 0.025) and improvement of the Birmingham IBS score (median 0 ± 1 vs 4 ± 1, *p* = 0.025) [134]. In another randomized, double-blind, controlled, prospective study including 71 children with IBS, treatment with inulin (900 mg, twice a day for 4 weeks) was less effective than probiotics and synbiotics [135]. In 2017, Shulman and colleagues found that the psyllium fiber (6 g for ages 7–11 years and 12 g for ages 12–17 years, for 6 weeks) lessened the mean number of pain episodes compared to placebo (8.2 ± 1.2 vs 4.1 ± 1.3, *p* = 0.03) in a randomized, double-blind trial, including 84 children with IBS [136]. Recently, Menon and colleagues performed a double-blind randomized controlled trial in which 43 children with IBS were assigned to receive psyllium and 38 placebo. Four weeks of psyllium supplementation significantly reduced IBS severity scoring scale (IBS-SSS) [75 (42.5–140) vs 225 (185–270); *p* < 0.001] and showed a higher remission rate (IBS-SSS < 75) compared to placebo (43.9% vs 9.7%, *p* < 0.0001) [137].

A recent systematic review and meta-analysis [138] evaluated the results of the previously discussed four studies [134–137] to assess the effects of hydrophilic fiber supplementation in children with IBS. Data, resulting by very low or low evidence, suggested that fiber supplements is efficacious in improving abdominal pain in children with IBS.

PICO 13: Should probiotics be used to treat global IBS symptoms in children?

Statement: We recommend the use of certain probiotic strains to treat global IBS symptoms.

Statement endorsed, overall agreement: 88.9%: A + 50%, A 38.9%, A- 11.1%, D- 0%, D 0%, D + 0%.

LoE: Moderate; SOR: Conditional.

Summary of evidence: The use of probiotics in the treatment of children with IBS has been the subject of several clinical trials and systematic reviews with meta-analysis [139, 140].

*L. rhamnosus* GG has the largest data (4 RCTs) [141–144] showing a significant reduction of the frequency and intensity of abdominal pain in children with IBS compared to the placebo group. The daily dose of *L. rhamnosus* ranged from 1 × 10^9^ CFU twice a day to 1 × 10^10^ CFU once a day.

A meta-analysis of the first three RCT [141–143] on the efficacy of *Lactobacillus* GG for the treatment of abdominal pain-related functional gastrointestinal disorders in childhood found that LGG treatment significantly reduced intensity and frequency of pain in the IBS subgroup (3 RCTs, *n* = 167; RR = 1.70, 95% CI 1.27–2.27, number needed to treat [NNT] 4, 95% CI 3–8) [145]. Only one RCT evaluated the effect of *L. reuteri* DSM 17938 in children with IBS. Children in the intervention group had significantly more days without pain (median 89.5 vs 51 days, *p* = 0.029) and less severe abdominal pain during the 2nd month (*p* < 0.05) and the 4th month of the intervention (*p* < 0.01). Noteworthy, the 2 groups did not differ in the duration of abdominal pain, stool type, or absence from school [146].

In a triple arm RCT in children with IBS, probiotic and synbiotic treatment based on *Bifidobacterium lactis* B94 (5 × 10^9^ CFU) improved belching-abdominal fullness (*p* < 0.001), bloating after meals (*p* = 0.016), and constipation (*p* = 0.031) compared to prebiotic (inulin) [135].

The same RCT investigated the efficacy of synbiotic (*Bifidobacterium lactis* B94 with inulin), probiotic (*B. lactis* B94), and prebiotic (inulin) treatment in pediatric IBS [135]. This study included 71 children between the ages of 4 and 16 years who were diagnosed with IBS according to Rome III criteria. Probiotic treatment improved belching-abdominal fullness (*p* < 0.001), bloating after meals (*p* = 0.016), and constipation (*p* = 0.031).

The efficacy of *Bacillus coagulans* Unique IS2 was evaluated in double-blind RCT involving children with IBS [147]. A total of 141 children in the age group 4–12 years, diagnosed with IBS according to Rome III criteria, were enrolled. Children received either *B. coagulans* Unique IS2 chewable tablets or placebo once a day for 8 weeks followed by a 2-week follow-up period. The *B. coagulans* Unique IS2 treated group showed a greater reduction in pain scores as evaluated by a weekly pain intensity scale. There was a significant reduction (*p* < 0.0001) in pain intensity in the probiotic treated group (7.6 ± 0.98) as compared to the placebo group (4.2 ± 1.41) by the end of the treatment period (8 weeks). There was also a significant improvement in stool consistency as well as a reduction in abdominal discomfort, bloating, staining, urgency, incomplete evacuation and passage of gas. Bowel habit satisfaction and global assessment of relief was also observed in the *B. coagulans* Unique IS2 treated group, compared to the placebo group [147].

A recent RCT assessed the efficacy and safety of adding *B. clausii* versus placebo to conventional treatment of pediatric IBS in Mexico [148]. Symptoms improvement, reported complete symptoms relief, stool evaluations, bloating, abdominal pain/intensity, and IBS behaviour were similar between groups.

The efficacy of selected mixture of probiotics has also been explored in children with IBS.

A multicenter international double-blinded, placebo-controlled, cross-over RCT examining the effect of a probiotic mixture of 8 different strains (*Lactobacillus acidophilus*, *Lactobacillus plantarum*, *Lactobacillus casei*, *Lactobacillus bulgaricus*, *Bifidobacterium breve*, *Bifidobacterium longum*, *Bifidobacterium infantis* and *Streptococcus thermophilus*) was conducted in children and adolescents with IBS [149]. The results showed that the probiotic group experienced a significant reduction in the frequency and intensity of abdominal pain over placebo. Likewise, significant improvement of bloating/gassiness and relief of symptoms as well as caregivers’ satisfaction was reported whilst stool pattern did not change during the period of intervention. Furthermore, the efficacy of probiotic mixture of three Bifidobacteria (*Bifidobacterium infantis* M-63, *B. breve* M-16V, and *B. longum* BB536) was assessed in a RCT including 48 children with IBS [150]. This study showed a significantly higher proportion of children with complete resolution of abdominal pain (*p* = 0.006) and a significant reduction of pain frequency (*p* = 0.02) as well as a significantly higher quality of life in children with IBS treated with probiotics when compared to the placebo group.

A recent ESPGHAN position paper on probiotics for the management of pediatric gastrointestinal disorders [151] included a weak recommendation, with a moderate certainty of evidence, for the use of *L. rhamnosus* GG (at a dose of 1 × 10^9^ CFU to 3 × 10^9^ CFU twice a day) in the reduction of pain frequency and intensity in children with IBS. Interestingly, a meta-analysis published in 2021 including seven RCTs with 441 participants, showed a higher effect of probiotic supplementation in patients under 10 years old (WMD =—2.55; 95% CI—2.84 to—2.27) compared to patients aged 10–18 years (WMD =—1.70; 95% CI—2.18 to—1.22). Moreover, the length of supplementation longer than 4 weeks was more effective (WMD =—2.43; 95% CI—2.76 to—2.09) [139].

PICO 14: Should polyethylene glycol be recommended to treat constipation in children with IBS-C?

Statement: We recommend to use PEG to treat constipation in children with IBS-C.

Statement endorsed, overall agreement: 100%: A + 100%, A 0%, A- 0%, D- 0%, D 0%, D + 0%.

LoE: unable to assess with GRADE; SOR: Consensus recommendation.

Summary of evidence: Polyethylene glycol (PEG) is the recommended treatment for functional constipation in infants (older than 6 months) and children [152, 153]. However, its effects on symptoms of IBS still need to be assessed. PEG is an osmotic laxative, that causes increased water retention and increased osmotic pressure in the lumen of the colon by binding to water molecules. As a result, the stool softens, and bowel movements occur more frequently [154].

Only one RCT evaluated the efficacy and safety of PEG 3350 + electrolytes (E) vs placebo in adult patients with IBS-C. Spontaneous complete bowel movements, responder rates, stool consistency and severity of straining showed superior improvement in the PEG 3350 + E group over placebo at week 4 [155]. The most common drug related adverse events were abdominal pain (PEG 3350 + E, 4.5%; placebo, 0%) and diarrhea (PEG 3350 + E, 4.5%; placebo, 4.3%).

No RCT has been performed in children. However, it is worth noting that functional constipation in children may indeed lead to an erroneous diagnosis of IBS-D or IBS-M. In a small study including 42 children, a decrease of more than 50% in abdominal pain intensity (and resolution of diarrhea) was observed in 8 (80%) and 14 (87%) patients with IBS-D and IBS-M. The effect on abdominal pain was not significantly different from that observed in 15 (93%) IBS-C children, considered as control group [63].

PICO 15: Should secretagogues be used to treat IBS-C symptoms in children?

Statement: We recommend against the use of intestinal secretagogues for the treatment of pediatric IBS-C.

Statement endorsed, overall agreement: 94.4%: A + 83.3%, A 11.1%, A- 5.6%, D- 0%, D 0%, D + 0%.

LoE: unable to assess with GRADE; SOR: Consensus recommendation.

Summary of evidence: Lubiprostone (a prostaglandin E1 derivative that stimulates type 2 chloride channels and promotes intestinal chloride fluid secretion) and linaclotide (a guanylate cyclase C agonist that activates intestinal secretion of chloride and bicarbonate iones) have demonstrated efficacy in adult patients with IBS-C. However, lubiprostone did not show a significant benefit over placebo in children with functional constipation [156]. No RCT has been published in children with IBS.

Only a retrospective chart review has been reported on the use of linaclotide in pediatric IBS [157]. This study included 33 children [median age 15.8 years (IQR 14.7–16.7)] treated with linaclotide (72–290 μg a day) for IBS-C. Over 40% of patients with IBS-C had a positive clinical response at a median follow-up of 2.4 months after starting linaclotide. Approximately, 30% of patients experienced adverse events and 27% stopped using linaclotide due to adverse events. The most common adverse events were diarrhea, abdominal pain, nausea, and bloating.

Further prospective controlled studies are needed to confirm these findings and to identify which patients are most likely to benefit from linaclotide.

PICO 16: Should 5-HT4 agonists be used to treat IBS-C symptoms?

Statement: We suggest against the use of 5-HT4 agonists in pediatric patients with IBS-C.

Statement endorsed, overall agreement: 100%: A + 77.8%, A 22.2%, A- 0%, D- 0%, D 0%, D + 0%.

LoE: Low; SOR: Conditional.

Summary of evidence: 5-Hydroxytryptamine-4 (5-HT4) receptor agonists enhances motility in the gastrointestinal tract by stimulating serotonin release [158]. A number of 5-HT4 agonists have been developed for the treatment of IBS-C and FC in adults, with tegaserod and prucalopride currently approved for the treatment of IBS-C in USA and of chronic idiopathic constipation both in USA and in Europe, respectively.

Lack of selectivity for the 5-HT4 receptor has limited the clinical success of tegaserod. Because of potential cardiovascular side effects, tegaserod is currently licensed in USA (not in Europe) only for the treatment of IBS-C in females aged 65 years old and younger without a history of ischemic cardiovascular disease.

Tegaserod is not approved for pediatric use. The only RCT performed in pediatrics involved 48 adolescents (13–18 years) with IBS-C randomly allocated to polyethylene glycol 3350 (PEG 3350) or combination therapy consisting of PEG 3350 and tegaserod. After 4 weeks of treatment, although both groups experienced a significant increase in the frequency of bowel movements, a significant reduction in pain level was only experienced by children receiving tegaserod in combination with PEG 3350 (21 patients). No adverse effects were reported [159].

Prucalopride, compared with other 5-HT4 receptor agonist, has a high selectivity for the receptor, thus minimizing the potential cardiac side effects and exhibiting a favorable safety [160]. A meta-analysis published in 2019 including 8 RCTs showed that prucalopride was more effective than placebo in treating adults with chronic idiopathic constipation [161]. Although an open-label, non-comparative study demonstrated an apparent favorable efficacy in children with constipation [162], the only pediatric randomized, placebo-controlled, double-blind, phase 3 trial designed to evaluate the efficacy of prucalopride in 213 children (6 months to 18 years old) with functional constipation (106 prucalopride, 107 placebo) showed that the proportion of responders was similar between groups [163]. To date no RCTs have been conducted on prucalopride in pediatric patients with IBS-C.

PICO 17: Should rifaximin be used to treat global IBS symptoms?

Statement: The use of rifaximin could be considered in children with IBS without constipation in which other treatments have failed.

Statement endorsed, overall agreement: 94.5%: A + 55.6%, A 38.9%, A- 5.6%, D- 0%, D 0%, D + 0%.

LoE: very low; SOR: Consensus recommendation.

Summary of evidence: Rifaximin is a semisynthetic broad-spectrum and poorly absorbable antibiotic derived from rifamycin. Being not absorbed from the gastrointestinal tract, rifaximin is mainly used to treat infectious diarrhea caused by non-invasive pathogens. Small intestinal bacterial overgrowth (SIBO) has been proposed to underlie the symptoms of IBS in some cases, therefore the use of rifaximin has been proposed to counteract gut dysbiosis [164]. Although data from adult literature support the beneficial effect of rifaximin on IBS without constipation, available data on the effects of rifaximin in children with IBS are scant and conflicting. The only double-blind placebo-controlled trial failed to demonstrate superiority of rifaximin over placebo [165]. In this RCT, the efficacy of rifaximin was evaluated in 75 children (8–18 years) with chronic abdominal pain (CAP) defined according to Rome II criteria. Patients were randomized in a 2:1 ratio, double-blind fashion, to receive a 10-day course of 550 mg of rifaximin or placebo 3 times a day. Subjects underwent lactulose hydrogen/methane breath test (LBT) to diagnose SIBO and completed symptom-based questionnaires at baseline and 2 weeks after treatment. Forty-nine children received rifaximin (26 IBS, 8 functional dyspepsia, 15 functional abdominal pain) and 26 received placebo. SIBO was documented in 94% of patients who received rifaximin and 92% who received placebo. There was no significant difference in symptom improvement between groups; moreover, only 20% of children treated with rifaximin achieved a normalization of repeat LBT. The authors concluded that, similar to adults with IBS, the prevalence of SIBO in children with CAP is high, but treatment with 10 days of rifaximin has low efficacy in normalizing LBT.

Another prospective pediatric study assessed the effects of rifaximin treatment (600 mg a day for 1 week) on SIBO and gastrointestinal symptoms in 50 consecutive children with IBS (age 9.9 ± 3.7 years) [166]. All subjects underwent LBT before and one month after the investigational treatment, while symptoms were assessed at baseline and one month after treatment. The prevalence of abnormal LBT was 66%. LBT normalization rate was 64%. Symptom score, significantly higher in IBS patients with SIBO, significantly improved after treatment with rifaximin.

Double blind placebo-controlled interventional studies are warranted in children with IBS to verify the real impact of SIBO on gastrointestinal symptoms and to evaluate the effect of rifaximin in pediatric IBS.

PICO 18: Should loperamide be used to treat IBS-D symptoms?

Statement: We recommend the use of loperamide to manage diarrhea in IBS-D, although its chronic use must be avoided.

Statement endorsed, overall agreement: 100%: A + 18.8%, A 81.2%, A- 0%, D- 0%, D 0%, D + 0%.

LoE: unable to assess with GRADE; SOR: Consensus recommendation.

Summary of evidence: Loperamide is a synthetic opioid that, by acting on opiate receptors in the myenteric plexus of the large intestine, inhibits peristalsis, prolongs transit time, decreases fecal volume. It is used as an anti-diarrheal agent available as over-the-counter medication for treating diarrhea. A meta-analysis of RCT showed that loperamide can be considered safe and effective in treating acute diarrhea in children older than 3 years since deaths have been reported in young children [167]. Data from RCTs performed in adults suggest a potential benefit in treating symptoms of IBS-D, but the risk of adverse events (especially prolonged QTc), tachyphylaxis and poor tolerability limits the chronic use of the drug [19].

To date, no studies have been conducted on loperamide in pediatric patients with IBS.

PICO 19: Should antispasmodics be used to treat global IBS symptoms?

Statement: The use of antispasmodics could be considered for global symptom improvement in children with IBS when other therapeutic strategies have failed.

Statement endorsed, overall agreement: 100%: A + 55.6%, A 44.4%, A- 0%, D- 0%, D 0%, D + 0%.

LoE: very low; SOR: Consensus recommendation.

Summary of evidence: Antispasmodics are a heterogeneous group of medications that suppresses smooth muscle contractions in the gastrointestinal tract. Direct smooth muscle relaxants (e.g., papaverine, mebeverine, peppermint oil), anticholinergic agents (e.g., butylscopolamine, hyoscine, cimetropium bromide, pirenzepine) and calcium channel blockers (e.g., alverine citrate, otilonium bromide, pinaverium bromide) have been used to treat IBS for decades with the rationale that a subgroup of patients have abnormal GI contractility (spasms) that results in pain and altered bowel habit [168]. Although the exact pharmacological mechanism of these agents is not always clear, data coming from RCTs comparing antispasmodics to placebo or other treatments have showed consistently a positive effect of these drugs in relieving IBS symptoms in adults [169]. Therefore, recent Italian guidelines of IBS recommend for the use of antispasmodics for global symptoms improvement in patients with IBS [19].

Although antispasmodics are extensively used in clinical practice for treating childhood IBS, there are no meta-analyses and only 2 RCTs have been conducted in paediatric population [170].

The efficacy of trimebutine maleate has been evaluated in a RCT (vs no treatment) on 78 children with IBS (Rome III criteria). After 3 weeks, symptoms relief was observed in the 95% of trimebutine maleate (3 mg/kg/day, 3 times a day) group compared with 20.5% who had spontaneous recovery. No drug related side effects were observed [171].

Later, in a RCT (vs no treatment) conducted in patients with IBS, including adolescents (from 15 years of age), 6 weeks of both trimebutine (100 mg twice a day) and mebeverine (135 mg twice a day) resulted in a statistically significant improvement of symptoms without differences in terms of efficacy among the treatment groups. None of the patients experienced side effects [172].

In a 2-week blinded RCT on 42 children (8–17 years of age) with IBS, the treatment with enteric coated pH-dependent release formulation of peppermint oil (patients > 45 kg received 2 peppermint oil capsules and smaller children 1 capsule 3 times a day; each capsule contained 187 mg of peppermint oil) resulted in a significant improvement in the severity of IBS symptoms compared with placebo (arachis oil) (76% vs 19%; *p* < 0.001). The study did not report any side effects [173].

In conclusion, though existing evidence suggests that antispasmodics may be used to treat global IBS symptoms in adults (with a good safety profile), physicians should be aware of the heterogeneity of the studies, both in terms of interventions and outcome measures [19]. Although there is currently no strong evidence to support the use of antispasmodics to treat IBS symptoms in children, based on anecdotal paediatric data and adult experience, antispasmodic drugs can be prescribed to aim at symptoms improvement in children with IBS when other therapeutic strategies have failed.

PICO 20: Should gut-brain neuromodulators be used to treat IBS symptoms?

Statement: The use of gut-brain neuromodulators, under specialist supervision, could be considered to treat severe abdominal pain in children with IBS in which other treatments have failed.

Statement endorsed, overall agreement: 94.4%: A + 50%, A 44.4%, A- 5.6%, D- 0%, D 0%, D + 0%.

LoE: Moderate; SOR: Strong.

Summary of evidence: In children with IBS, the benefits of antidepressants are controversial since data are conflicting. A recent Cochrane review evaluated the current evidence for the efficacy and safety of antidepressants for FAPDs in children and adolescents [174]. The authors found two trials in children with IBS, both using amitriptyline, and concluded that the current evidence to support the routine use of antidepressants in children with IBS is limited.

The first randomized double-blind placebo-controlled trial on amitriptyline in pediatric IBS included 33 patients (aged between 12–18 years old; 24 females), 17 of them received placebo and 16 amitriptyline [175]. A significant improvement in overall quality of life, abdominal pain and IBS-associated diarrhea, was observed in the amitriptyline group compared to the placebo one. This study is difficult to interpret, due to incomplete information regarding abdominal pain scores. Moreover, patients in the placebo group responded with worsening of pain.

The first multicenter randomized placebo-controlled trial on patients with FGIDs (IBS, FAP, FD), failed to identify any significant differences between amitriptyline and placebo in terms of improvement of overall symptoms [176]. Ninety children were enrolled and 83 completed the study, with 40 children randomized in the placebo arm and 43 in the amitriptyline arm. Both groups showed a significant improvement in pain scores after 4 weeks, but no differences were found between the groups (intention-to-treat analysis: *p* = 0.81; per-protocol analysis: *p* = 0.83, NS respectively). The only significant result was observed in term of reduction of anxiety scores among patients treated with amitriptyline (*p* < 0.0001), whilst no differences between the groups were found regarding depression coping. In this study, the large placebo response (57.5%) may have contributed to the negative results.

A retrospective study evaluated the response rate to tricyclic antidepressant in pediatric IBS. On a total of 55 patients treated with antidepressants, the overall response rate was 82.4% (84.4% with amitriptyline and 78.9% with imipramine), but this study did not include a placebo control group [177].

The efficacy of amitriptyline was recently evaluated in pediatric FAPD patients diagnosed according to Rome IV criteria [178]. In this open-label trial, children were randomized to amitriptyline or placebo for 12 weeks. There was a significant difference in pain improvement in terms of reduction in scores for intensity (3.4 vs 0.9), frequency (3.6 vs 0.6), duration (3.5 vs 0.9), and QoL (2.3 vs 0.9) between amitriptyline and placebo group (*p* < 0.001 in all). Interestingly, pain scores showed significant improvement for IBS and functional abdominal pain not otherwise specified (FAP-NOS) but not in FD in the amitriptyline group compared with placebo. Minor adverse events were comparable between the groups (25.3% vs 13.5%, respectively, *p* = 0.07). Interestingly, after discontinuation of amitriptyline, 70% had sustained response over a mean follow up of 15.84 ± 5.6 months.

In conclusion, further studies with adequate sample size, homogenous and relevant outcome measures and longer follow up are needed in pediatric patients with IBS to determine if antidepressant are more effective than a placebo.

It should be noted that antidepressants have an FDA black box warning, due to increased risk of suicidal thoughts and ideation [179]. The prevalence of major psychiatric conditions in children reporting chronic abdominal pain is low and there are currently no published reports of increased suicidal behavior in non-depressed children receiving low dose of amitriptyline for the treatment of FAPDs, but prescription under specialist supervision is warranted.

If from one perspective the high placebo effect in patients with FAPDs emphasizes the need of a biopsychosocial approach, from the opposite perspective it might undervalue the potential efficacy of a therapeutic interventions. For instance, in the study from Saps and colleagues the large placebo response (57,5%) may explain the negative results [176]. In a meta-analysis of 21 trials of childhood FAPD, 41% of patients with abdominal pain-related FGIDs improved on placebo (95% CI 34–49; 17 studies) and 17% reported no pain (95% CI 8–32; 7 studies) [180]. The high placebo effect in children and adolescents with FADPs was recently confirmed in a recent multicenter crossover RCT conducted in 3 US centers. In this study, the authors evaluated the efficacy of open-label placebo (OLP) in children and adolescents with FAP (*n* = 16—53.3%) or IBS (*n* = 14—46.7%) defined according to Rome III criteria, showing a significant lower mean (SD) pain scores during OLP treatment compared with the control period (39.9 [18.9] vs 45.0 [14.7]; difference, 5.2; 95% CI, 0.2–10.1; *p* = 0.03). Interestingly, during OLP, patients with functional abdominal pain or irritable bowel syndrome took significantly fewer pain medications. This study suggests that open-label placebo may be an effective treatment for children and adolescents with functional abdominal pain or irritable bowel syndrome [181].

In conclusion, although further studies with adequate sample size, homogenous and relevant outcome measures and longer follow-up are needed in children with IBS, the use of tricyclic antidepressants under close specialist supervision (e.g. pediatric gastroenterologist or child psychiatrist), could be considered in those patients with severe symptoms significantly affecting the quality of life and with significant psychological comorbidities. The treatment should be started only after adequate counseling provided to the patient and family.

PICO 21: Should complementary alternative therapies be used to treat IBS symptoms?

Statement: The use of certain complementary alternative therapies could be considered to treat IBS symptoms.

Statement endorsed, overall agreement: 100%: A + 50%, A 50%, A- 0%, D- 0%, D 0%, D + 0%.

LoE: very low; SOR: Conditional.

Summary of evidence: Yoga can be considered as a form of behavioural therapy and consists of general relaxation exercises, breathing exercises, focused training for abdominal relaxation and positive reinforcement by focusing thoughts on a single topic and good experiences.

In an uncontrolled pilot study, 20 children aged between 8–18 years, with IBS or FAP, received 10 yoga sessions and also practiced at home. Pain frequency was significantly decreased at the end of therapy compared to baseline. In the 8–11 years group, pain intensity was also significantly decreased. Interestingly, after 3 months there still was a significant decrease in pain frequency in the younger patient group. Parents reported a significantly higher QoL after yoga treatment. This pilot study suggests that yoga exercises are effective for children with FAP and IBS, resulting in significant reduction of pain intensity and frequency, especially in children of 8–11 years old [182].

A RCT compared the benefits of Iyengar yoga (IY) with standard care in 51 patients with IBS and found that, when compared to controls, young adults (18–26 years) assigned to the yoga group reported significant improvement of IBS symptoms, disability, psychological distress, sleep quality, and fatigue, but only adolescents (14–17 years) assigned to yoga group reported also a significant improvement in physical functioning [183]. These findings suggest that a brief IY intervention is a feasible and safe adjunctive treatment for young people with IBS. The age-specific results suggest that yoga interventions may be most fruitful when developmentally tailored.

These results were confirmed by another RCT study comparing YT to standard medical therapies (SMT) in 69 children (35 children were allocated to YT and 34 children to SMT). YT was significantly more effective than SMT in decreasing both the frequency and the intensity of pain at 12-months follow-up [184]. These findings suggest that a brief IY intervention is a feasible and safe adjunctive treatment for young people with IBS. The age-specific results suggest that yoga interventions may be most fruitful when developmentally tailored. However, a recent systematic review and meta-analysis evaluated the current evidence for the efficacy of various psychosocial interventions, including Yoga, in the management of FAPDs in children and adolescents, no difference in treatment success was found when comparing yoga with no interventions (RR, 1.09; 95% CI, 0.58–2.08) [185].

The effects of the Benson relaxation technique on the severity of symptoms and quality of life in Iranian children have been evaluated in a quasi-experimental study. The Benson relaxation technique was implemented for 3 weeks for the experimental group, while the control group only received the typical medical therapy with no special intervention. The mean score of symptom severity in children with IBS was 13.88 in the experimental group, which changed to 9.83 in the post-test, indicating a significant difference (*p* < 0.000). The pre-test and post-test mean scores for quality of life in this group were 118.94 and 102.77, respectively, indicating a significant difference (*p* < 0.001). This study suggests that the Benson relaxation technique can be a non-pharmacological solution to reduce the severity of symptoms and improve the quality of life of children with IBS [186].

Although pilot pediatric studies on yoga yielded good results, larger well-designed trials with appropriate comparative groups are needed to establish the effect of yoga and other complementary and alternative therapies.

PICO 22: Should psychologically directed therapies be used to treat global IBS symptoms?

Statement: We strongly recommend the use of psychologically directed therapies for the treatment of global symptoms.

Statement endorsed, overall agreement: 94.4%: A + 88.9%, A 5.6%, A- 5.6%, D- 0%, D 0%, D + 0%.

LoE: Low; SOR: Strong.

Summary of evidence: Different psychologic interventions, such as cognitive behaviour therapy (CBT) and hypnotherapy (HT), have shown efficacy in children with IBS which might persist many years after cessation of therapy [187, 188]. CBT is a primary psychotherapy that aims to decrease anxiety and modify awareness, emotion and reaction to stress related to somatic symptoms, whilst in HT children are induced into a deep state of relaxation so they can be guided to learn how to control their gut function by modifying their experiences, perceptions, feelings and thoughts.

A recent systematic review and meta-analysis evaluated the current evidence for the efficacy of various psychosocial interventions, such as CBT, HT and educational support in the management of FAPDs in children and adolescents [185]. A total of 12 studies, including 785 children and adolescent, compared CBT with no interventions, and a total of 5 studies compared CBT with educational support. When compared to no intervention, the authors found moderate evidence that CBT was associated with higher success in treatment (*n* = 324 children; RR: 2.37; 95% CI 1.30–4.34; NNT = 5) as well as with a decrease in both frequency (*n* = 446 children; RR: -0.36; 95% CI, -0.63 to -0.09) and intensity (*n* = 332 children; RR: -0.58; 95% CI, -0.83 to -0.32) of pain. When compared to educational support, there was no difference in the success rate between the two psychosocial approaches (*n* = 127 children; MD: − 0.36; 95% CI, -0.87 to 0.15). Similarly to CBT, HT is associated to a limited but significant higher success than no intervention (RR: 2.86; 95% CI, 1.19–6.83; NNT = 5).

A randomized controlled study on the treatment of children with FAP or IBS with gut-directed hypnotherapy (HT) (IBS; *n* = 22) or standard medical treatment (SMT) (*n* = 23) showed that HT significantly reduced pain scores, compared to SMT (*p* < 0.001) [187]. Interestingly, in a long-term (mean duration of 4.8 years) follow-up study of the same population, HT was still superior to SMT with 68 vs 20% of the patients in remission (*p* = 0.005). Pain intensity and pain frequency scores at follow-up were 2.8 and 2.3, respectively, in the HT group compared with 7.3 and 7.1 in the SMT group (*p* < 0.01). Also, somatization scores were lower in the HT group (15.2 vs 22.8; *p* = 0.04). No differences were found in QoL, doctors’ visits, and missed days of school or work between the two groups [188].

Although the reported long-lasting beneficial effects of psychological interventions in children with IBS make them a highly valuable therapeutic option, one of the main disadvantages of behavioural interventions is that psychologists might not be readily available and costs could be high. To overcome these problems, studies have shown that internet/audio-delivered HT and cognitive behavioural therapy (CBT) can be just as effective in children with IBS [189, 190].

The effectiveness of HT by means of home-based self-exercises using a CD was compared with that of individual HT (iHT) performed by qualified therapists [189]. In this non-inferiority randomized clinical trial, conducted in a tertiary care center throughout the Netherlands, 126 children (aged 8–18 years) with IBS were included in the study. After 1-year follow-up, the 62.1% treatment success in the CD group was non-inferior to the 71.0% in the iHT group (difference, -8.9%; 90% CI, -18.9% to 0.7%; *p* = 0.002).

Another RCT evaluated the efficacy of internet-delivered cognitive behaviour therapy (Internet-CBT) in adolescents with IBS [190]. In this study, 101 adolescents (13–17 years of age) fulfilling the Rome III criteria for IBS were randomized to either Internet-CBT or a wait-list control. Dropout rates were low (6%) and all randomized patients were included in intent-to-treat analyses based on mixed effects models. Data showed a significant larger change before and after treatment in the total score of Gastrointestinal Symptoms Rating Scale for IBS (GSRS-IBS) (*p* = 0.006) and in quality of life and parent-rated gastrointestinal symptoms for the Internet-CBT group compared with the control group. After 6 months, the results were stable or significantly improved.

## Conclusions

IBS prevalence in children has increased in the last decade, significantly impacting on lives of affected patients and their families. Given the little knowledge about the physiopathology underlying IBS, unnecessary tests and medications are frequently performed and prescribed. Despite the lack and methodological limitations of the evidence, our group of experts deemed as essential to provide, for the first time in Italian literature, indications and recommendation, aiming to guide clinicians in the diagnosis and management of IBS in children. In the absence of a definitive laboratory biomarker or radiological diagnostic test, IBS remains a clinical diagnosis achieved according to Rome IV criteria and not as a result of an exclusion algorithm. Doubtless, alarm signs and symptoms’ must be investigated and serology testing for celiac disease is recommended. Only in patients with diarrhea, fecal calprotectin and C-reactive protein must be performed. In absence of alarm signs or symptoms, additional tests both invasive (endoscopy) or not invasive (testing for enteric pathogens, allergy/intolerance, SIBO) are not recommended. Management of children with IBS may include either non-pharmacological (diet, psychologic interventions, specific fibers and probiotics) and pharmacological strategies (PEG, rifaximin, antispasmodics, gut-brain neuromodulators), as long as they are individualized to the patient’s symptoms, often entailing a multidisciplinary approach.

## Data Availability

Not applicable.

## Abbreviations

IBS: Irritable Bowel Syndrome
DGBI: Disorder of gut-brain interaction
SIGENP: Italian Societies of Gastroenterology, Hepatology and Pediatric Nutrition
SIP: Italian Society of Pediatrics
SIGE: Italian Society of Gastroenterology and Endoscopy
SINGEM: Italian Society of Neurogastroenterology and Motility
GRADE: Grading of Recommendations, Assessment, Development, and Evaluation
SOR: Strength of recommendation
PICO: Patient, intervention, control, and outcome
RCT: Randomized controlled trials
IBS-C: Irritable bowel syndrome with predominant constipation
IBS-D: Irritable bowel syndrome with predominant diarrhea
IBS-M: Irritable bowel syndrome with mixed bowel habits
CD: Celiac disease
FAPDs: Functional abdominal pain disorders
tTG: Tissue transglutaminase
FC: Fecal calprotectin
CRP: C-reactive protein
ERS: Erythrocyte sedimentation rate
SIBO: Small intestinal bacterial overgrowth
LBT: Lactulose/methane breath test
FGIDs: Functional gastrointestinal disorders
GHBT: Glucose hydrogen breath test
FODMAPs: Fermentable oligo-, di- and monosaccharides, and polyols
GFD: Gluten-free diet
MD: Mediterranean Diet
IBS-SSS: IBS symptoms severity score
QoL: Quality of Life
FRD: Fructose-restricted diet
LRD: Lactose-restricted diet
PHGG: Partially hydrolyzed guar gum
PEG: Polyethylene glycol
IQR: Inter-quartile range
5-HT4: 5-Hydroxytryptamine-4
IY: Iyengar yoga
CBT: Cognitive behaviour therapy
HT: Hypnotherapy
GSRS-IBS: Gastrointestinal symptoms rating scale for IBS
